# Moving forward, leaving the patients behind? A multilevel assessment framework for evaluating patient-centred, integrated care quality

**DOI:** 10.1007/s11136-025-04121-8

**Published:** 2026-01-14

**Authors:** Sonja Cassidy, Ove Lintvedt, Francis Odeh, Conceição Granja, Terje Solvoll

**Affiliations:** 1 Department of Strategic ICT, Western Norway Regional Health (Helse Vest IKT), Bergen, Norway; 2 Norwegian Centre for E-health Research, University Hospital of North Norway, Tromsø, Norway; 3 Faculty of Nursing and Health Sciences, Nord University, Bodø, Norway; 4 Department of Health and Social Care, Municipality of Bodø, Bodø, Norway; 5 Department of Community Medicine, UiT – The Arctic University of Norway, Tromsø, Norway

**Keywords:** Patient-centred integrated care, Quality measurement, Mental health, Patient-reportedoutcomes, Multimorbidity, Health service quality research

## Abstract

**Purpose:**

Many current care assessment frameworks prioritise clinical and organisational outcomes over patient perspectives. This study aimed to identify gaps in existing patient-centred assessment methods and to develop a multilevel framework aligning quality evaluation with patient-defined priorities across macro (policy), meso (organisational), and micro (individual) levels, and technological levels.

**Methods:**

We used a primarily qualitative design, conducting a literature review of patient-centred integrated care assessment studies and integrating these findings with a longitudinal case study that examined how the patient’s perspectives were documented across multiple health information systems, synthesising evidence on existing practices with insights into how patient perspectives are integrated and represented for a comprehensive understanding.

**Results:**

In total, 32 studies were included. The review revealed ongoing misalignment between systemic evaluation practices and patient-defined outcomes, particularly for individuals with complex physical and mental health needs. Minimal patient involvement in developing evaluation criteria reflected a disconnect between policy-level targets and individual patient well-being. This misalignment was echoed in the case study, which underscored that personal goals and non-clinical needs were often unrecorded, highlighting the gap between evaluation metrics and genuinely patient-centred care.

**Conclusion:**

Integrated care quality assessment remains misaligned with patient-defined outcomes. We propose Patient-Reported Integrated Measures (PRIMs) as a conceptual contribution. PRIMs complement existing Patient-Reported Outcome Measures (PROM) and Patient-Reported Experience Measures (PREM) by capturing multidimensional outcomes that matter to patients and ensuring evaluation aligns with their goals. Integrating PRIMs into health information systems and research agendas can realign care evaluation with evolving patient priorities, thereby reducing the risk of leaving patients behind in future healthcare reforms.

## Introduction

As global populations age, chronic conditions are increasingly prevalent [1, 2]. Individuals managing co-occurring physical and mental health challenges often face unmet social and behavioural health needs and disproportionately higher rates of disability and premature mortality due to underdiagnosed physical health issues within siloed care systems [3]. This burden underscores the need for patient-centred, integrated care. In this paper, we adopt The World Health Organization’s (WHO) European Office for Integrated Health Care Services*’* definition, integrated care is: *a concept bringing together inputs*,* delivery*,* management and organization of services related to diagnosis*,* treatment*,* care*,* rehabilitation and health promotion* [4]. *Patient-centred care* draws on the broader concept of person-centred care, which emphasises the importance of understanding each patient’s context, experiences and personal strengths, promoting their active involvement in decisions [5]. Together, patient-centred, integrated care is expected to improve patient-perceived quality, enhance access and continuity of services, and reduce fragmentation and inefficiencies across the system [6–9].

Patient-centred, integrated care process unfolds through a series of contacts between people and providers across the system level (macro), the professional and organisational level (meso), and the individual level (micro) [10, 11]. At the macro level, incorporating the patient’s view on what they value in their care into health policies through tools like patient-reported outcomes (PROs) for cross-national benchmarking can enhance system performance. At the meso level, patient feedback can guide quality improvement and interdisciplinary collaboration aligned with patient priorities. At the micro level, embedding what matters to the person in shared decision-making can individualise care plans and improve outcomes [2, 12].

Digital technologies, including electronic health records (EHRs) and patient portals, offer mechanisms to collect and use patient feedback for decision-making [13]. However, current evaluation frameworks that rely on aggregated data from health information systems tend to prioritise system-level metrics such as cost-effectiveness and clinical outcomes [14–16]. This misalignment can obscure improvement efforts, overlooking patients’ reported experiences and priorities [2, 17], while limited involvement in digital health assessment further marginalises patients’ voices [18]. Consequently, the patient is still often *left behind*.

The OECD’s PaRIS initiative has highlighted the need for patient-informed evaluation for people with chronic and complex conditions [7]. Patient-Reported Outcome Measures (PROMs), capturing patients’ health outcomes, and Patient-Reported Experience Measures (PREMs), assessing patients’ experiences with care processes, have become common at the micro level to systematically collect data on outcomes and experiences [19]. However, these tools are often criticised for not fully capturing patient priorities [20], limiting their meaningful application at meso and macro levels [21]. In addition, these conventional mechanisms alone rarely capture the nuanced and ongoing nature of integrated care [14, 16, 22, 23], and patient-reported feedback informing clinical decisions, care coordination, or system improvement remains limited [7]. This limitation is rooted in evaluation tools that inadequately capture the patient’s full life context, goals, and function.

Consistent with the WHO’s definition of Quality of Life (QoL), defined as *an individual’s perception of their position in life in the context of the culture and value systems in which they live and in relation to their goals*,* expectations*,* standards*,* and concerns* [24], and with the more recent Comprehensive Mental Health Action Plan 2013–2030, including factors such as disability, housing and employment [3], and the OECD’s Better Life Initiative offering a multidimensional well-being framework [25], this study adopts a broad understanding of patient-centred quality that spans goals, needs, priorities, function and life context.

Despite the recognised need for this comprehensive perspective on quality, a clear, standardised, and holistic mechanism is missing to systematically capture patient-centred, integrated care priorities and apply this information consistently across the care continuum. To address these challenges, we propose a holistic framework for evaluating patient-centred, integrated care grounded in the patient’s perspective, and introduce *Patient-Reported Integrated Measures (PRIMs)*, to complement existing PROMs and PREMs in evaluation frameworks. This approach aligns with Objective 4 of the WHO Comprehensive Mental Health Action Plan 2013–2030, which aims to strengthen the collection and use of patient-reported information in health information systems and research (Target 4.1 and 4.2) [3].

## Background

### Theoretical foundation for evaluating patient-centred integrated care

Healthcare evaluation serves multiple purposes, from assessing interventions to guiding service improvement [26–28]. While the use of indicators to measure performance is now standard [29], prevailing approaches tend to privilege clinical effectiveness and efficiency, narrowing attention to what is readily countable rather than what patients experience as quality [30, 31].

Extending patient involvement to the co-development and validation of patient-centred measures may help to address current gaps [20, 32]. Involvement is linked to better self-rated health outcomes; however, it requires careful implementation to avoid deepening socio-economic, ethnic, and disability-related health inequalities in QoL and service access [33–35]. This highlights the need for a more inclusive epistemology that seeks and values experiential and subjective patient knowledge [36]. Patients frequently emphasise autonomy, dignity, being heard and compassion, values shaped by personal, cultural and life-course contexts [37, 38]. However, formal assessment processes often undervalue this expertise [29, 39].

Interpretive approaches further suggest that understanding emerges through dialogue, reflexivity and attention to meaning rather than solely through predefined surveys, challenging established assumptions [40, 41]. Healthcare quality is dynamic, evolving along the patient’s journey, influenced by technical competence, communication, and care settings. Aspects most visible to patients, such as being respected or listened to, do seem to be absent from standard evaluation tools, leaving critical elements unmeasured. Finally, definitions of health themselves shape how quality is conceptualised, either narrowing or expanding its scope across clinical, functional, relational and social dimensions [42–44]. Taken together, these perspectives motivate an evaluation approach that integrates patient-defined priorities as structured knowledge, aligns them across macro, meso and micro levels, and remains open to interpretive insight where meaning is negotiated as well as measured.

### Representing the patient perspective at the policy level

Over the past two decades, institutions such as the OECD have led efforts to assess healthcare quality through frameworks that guide policymaking [21]. The *OECD Health System Performance Assessment (HSPA)* framework [14] incorporates various indicators: *structural indicators*, such as system capacity, *process indicators*, such as timeliness of care, and *outcome indicators*, such as mortality rates, and has recently added dimensions like equity and trust [45–47]. Yet, system-level assessments still tend to prioritise goals such as resource use over patient-defined priorities [21].

Countries such as Norway have aligned healthcare quality assessments with international trends. Norway’s National Healthcare Quality Indicator System emphasises transparency, patient safety, and shared decision-making [48, 49]. Instruments like PROMs and PREMs capture micro-level perspectives, but their macro-level integration remains limited because they rarely reflect broader life context or cross-setting coordination [2]. In addition, macro-level patient-centred indicators often rely on standardised datasets that improvements struggle to represent evolving and context-specific patient priorities [50], contributing to gaps in using patient experience data for actionable quality improvements [51–53]. Moreover, fragmented EHRs hinder macro-level evaluations, highlighting a persistent disconnect between systemic priorities, organisational goals, and the lived patient experience [35].

### Aligning organisational, clinical, and patient priorities

Care evaluation addresses organisational processes and outcomes and how care is coordinated across teams and settings. Balancing organisational goals with the needs of patients and healthcare professionals is often complicated by differing views on what constitutes quality care [54]. Healthcare professionals face growing pressure to deliver high-quality services, but there are concerns that implementing patient-reported measures may divert attention from direct care and disrupt workflows, challenging organisational effectiveness. They also emphasise the need for training to integrate these measures into clinical decision-making effectively [55]. These tensions can create resistance as professionals weigh patient needs against evaluation goals [12, 56].

Tools designed to enhance collaboration and service coordination are gaining traction. The Care Process Self-Evaluation Tool (CPSET) enables multidisciplinary teams to assess organisational processes such as communication, coordination and responsiveness to patient and family needs [57–59]. However, evaluating care across organisational boundaries remains challenging due to weak links between organisational and patient objectives, process measures and clinical outcomes. This weak linkage constrains alignment with system-level objectives, making it difficult to track whether quality-improvement efforts remain coherent across macro, meso, and micro levels [56, 60].

### The patient’s view on healthcare assessment

Micro-level care evaluation centres on individual patient experiences shaped by higher-level practices and policies. Evaluating care quality across the patient’s care journey has been shown to be useful [11]. However, many methods rely on retrospective snapshots, which may be misleading as patients’ health conditions and experiences change over time [61]. Additionally, PROMs and PREMs are often standardised for general populations [62].

Research shows patients’ perceived value of care goes beyond medical treatment, including emotional support, practical assistance, goal alignment, self-efficacy, and seamless care transitions [63–65]. International initiatives continue to explore broader patient-informed metrics [1]. However, meaningful patient involvement in care assessment still lags. Extending participation beyond feedback collection to include co-designing services and validating PROMs and PREMs could address current gaps [20, 32]. Yet, participation in evaluation is often hindered by fragmented processes, competing priorities, limited resources, and a lack of practical knowledge on how to meaningfully involve patients [20, 32].

### Integrating patient perspectives into care evaluation: the digital perspective

Technological innovation has enhanced patient-centred care by integrating patient-reported data into EHR systems, enabling a more holistic view of the patient’s care pathway and supporting service performance monitoring [13, 66, 67]. Electronic PROMs and PREMs, collected via digital questionnaires, facilitate near real-time capture within clinical workflows, and research indicates that patients prefer this electronic format [68, 69]. However, poor interoperability across HISs can lead to fragmented data silos, limiting comprehensive understanding of patient journeys and practical system-level evaluation [2]. In addition, unstructured patient-centred information often leaves providers without actionable insights, and ongoing debates on standardisation complicate the capture of subjective experiences within rigid frameworks [20, 56, 70].

Artificial intelligence (AI) offers the potential to link patient-reported and clinical data to identify patterns and personalise care, and to better align macro-level policies, meso-level operations, and micro-level patient feedback into cohesive decision support. However, realising this potential requires strong ethical and equity-sensitive design, transparency and explainability, privacy and trust safeguards, and continuous monitoring to ensure tools reflect patient priorities and to prevent health inequities.

## Methods

### Study design

This study employed a qualitative approach, synthesising structured descriptions of existing care evaluation practice with qualitative insights into how patient perspectives help achieve a comprehensive understanding. A literature review, conducted following the Joanna Briggs Institute (JBI) method for evidence synthesis [71] and the PRISMA Extension for Scoping Reviews (PRISMA-ScR) guidelines [72], identified current frameworks for assessing patient-centred care.

**Fig. 1:**
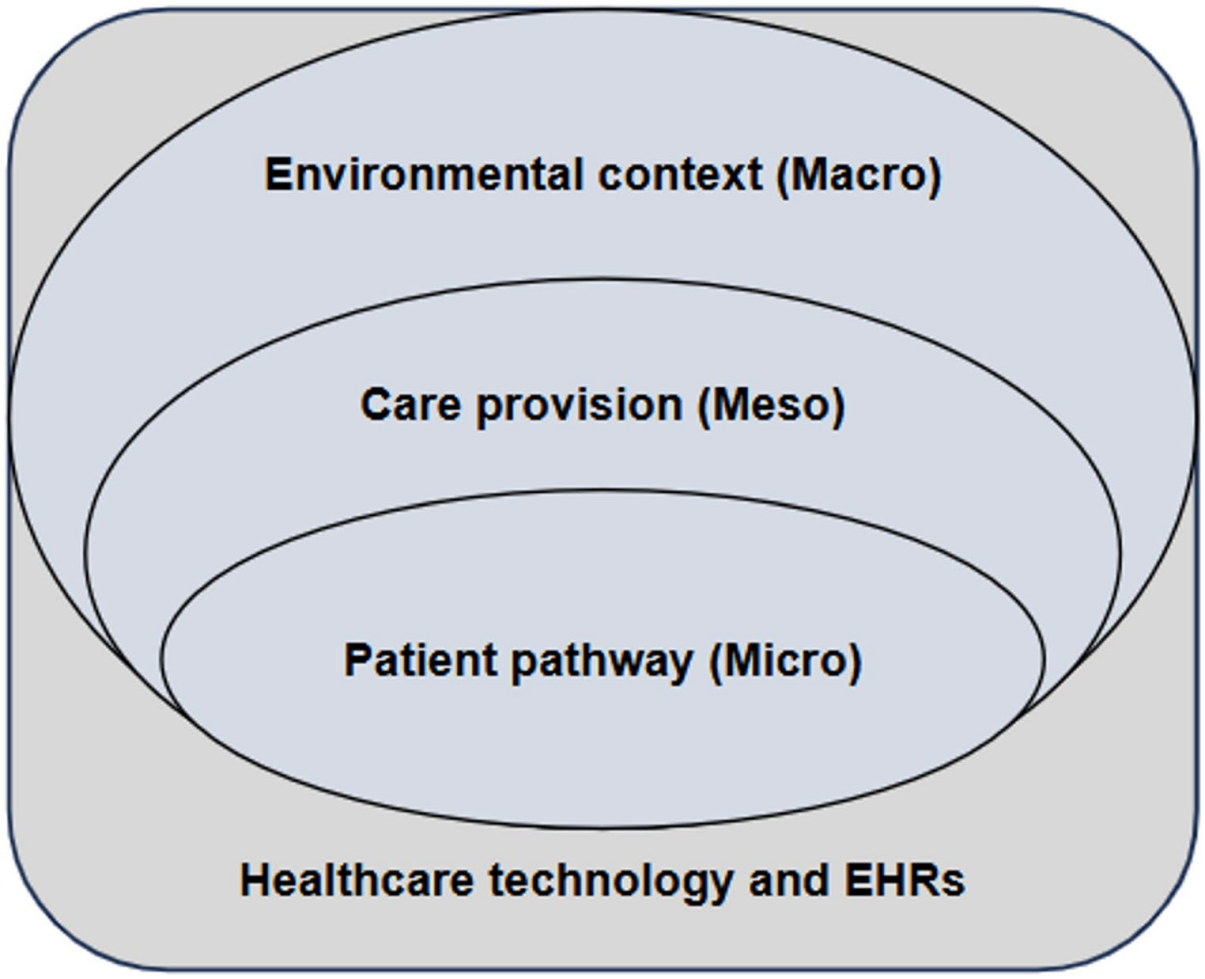
Conceptual model illustrating the healthcare system levels included in the PCICP framework. Adapted from Cassidy et al. [70]

Findings were initially categorised according to the five evaluation components described by Longworth et al. [28] (Participation, Experience, Context, Impact, and Alignment - PECIA) and a comparative deductive-inductive analysis was conducted based on the Patient-Centred Integrated Care Pathways (PCICP) framework [18]. PCICP is a multilevel pathway modelling framework that visualises the patient’s journey and links patient-defined goals, needs and priorities to clinical workflows, organisational routines, policy instruments, and the information systems that support care. The PCICP framework was selected due to its ability to integrate macro-level priorities, meso-level organisational goals, micro-level lived patient experiences and technological dimensions (Fig. 1).

To enrich the literature review, findings were compared with Cassidy et al.’s case study [70], which analysed longitudinal medical records and patient-provider interactions for individuals with co-occurring mental health conditions, such as anxiety and depression, and chronic physical diseases, such as cancer and cardiovascular disease. These records, collected over several decades, offered qualitative insights into how patient perspectives, such as personal goals, were documented across various care settings.

### Search strategy

The review followed the predefined protocol to ensure a systematic and transparent process [72, 73]. In line with PRISMA-ScR, the scope was defined using PCC: *Population*—patients receiving care across services; *Concept*—patient-reported information and the evaluation of patient-centred, integrated care; *Context*—macro, meso, micro and technological levels within health systems.

An initial exploratory search was conducted using Google Scholar to refine terminology and scope. Following team discussions, a formal search strategy was developed. The final systematic search was conducted in PubMed, Web of Science, and Google Scholar in February 2025. Results were de-duplicated using EndNote, and no other automation tools were used. The search was initially restricted to titles and abstracts to ensure relevance before progressing to full-text screening. Studies were included if they were open access, peer-reviewed, published in English between January 1, 2015, and January 1, 2025, and explicitly focused on patient-centred assessment in healthcare. Only open-access literature was included in the final review to ensure transparent and reproducible results. Google Scholar served as a supplementary source for backward and forward citation chasing. Results were sorted by relevance; approximately 350 records were screened. Consistent with the methodological framework for scoping reviews, a formal critical appraisal or risk-of-bias assessment of the included studies was not conducted. The primary aim of this review is to map the extent and nature of the literature, not to evaluate the quality of the evidence.

The search queries used variations of the keywords *healthcare quality*,* integrated care*,* patient-centred*,* participation*,* assessment*, and *outcome.* Grey literature was excluded to maintain consistency of peer-review across included studies.

All search strings are provided in Appendix A. The complete eligibility criteria are presented in Table 1.

**Table 1:** Eligibility criteria

Criterion	Inclusion	Exclusion
Publication type	Peer-reviewed, open access	Not peer-reviewed, not available via open access
Language	English	Non- English
Publication date	January 1, 2015, and January 1, 2025	Published outside this date range
Patient-centred focus	Explicit focus on patient-centred care assessment in healthcare	Studies focusing only on provider, organisational, or administrative perspectives, disease outcomes, medical treatments, or interventions not linked to care assessment
Level of care	Addressed multiple levels of care, such as primary, hospital, home/community care	Focused exclusively on a single setting or professional group, such as only hospital, only GPs, only nurses
Study type	Empirical or conceptual studies (quantitative, qualitative, mixed methods); includes theme-based outcome studies	Protocols for planned interventions with no findings and studies exclusively addressing patient involvement in service design
Assessment scope	Assessment of care processes, outcomes, impact, quality, or use of indicators or frameworks for evaluation	Studies limited to patient involvement in service or intervention co-design (not care assessment)
Methodological transparency	Describes specific assessment methods, measurement tools, or indicators	Describes care in general terms without a clear methodology for evaluation

### Data extraction

A structured spreadsheet was used to extract and document relevant components of patient-centred integrated care assessments. Data were extracted using a piloted form. Two reviewers (SC, OL) independently screened titles and abstracts and then full texts against the eligibility criteria. Discrepancies were resolved by consensus, with a third reviewer available to adjudicate when needed. Findings were categorised based on the evaluation components defined by Longworth et al. [28], including *Participation*, *Context*, *Experience*, *Impact* and *Alignment* (PECIA), which were then mapped to the macro, meso, micro and technological dimensions of the PCICP framework (Table 2).

**Table 2:** Patient-centred, integrated care assessment (PECIA) components

Healthcare dimensions/Evaluation components	Participation (*P*)	Experience (E)	Context (C)	Impact (I)	Alignment (A)
Macro	How patient perspectives are represented in national and regional policymaking, regulatory bodies, and large-scale healthcare initiatives	Aggregated or benchmarked patient-reported outcomes and experiences reflecting overall population-level satisfaction and perceived quality	The socio-political, economic, and cultural factors that influence system-wide care assessment such as national health priorities	The long-term effects of system-level policies on care quality, access, equity, and patient outcomes	The degree to which national policies, guidelines and performance indicators reflect and incorporate patient perspectives
Meso	How healthcare organisations involve patients in quality improvement initiatives	Patient-reported experiences with care delivery at the organisational level, including interactions with multidisciplinary teams, communication, and service delivery	Organisational culture, internal structures, resource allocation, and the interplay between professional values and patient views and expectations	The effect of patient-centred care assessment on patient care outcomes such as service coordination improvements	How well organisational practices, quality indicators, and evaluation frameworks align to reflect patient priorities and feedback
Micro	The level of individual patient involvement in care assessment planning, indicator development and care evaluation	Patient-reported experience and outcomes, such as the quality of interactions with healthcare professionals or psychological and physical health	Patient-specific factors such as demographics, health literacy, cultural background, and personal care goals	Individual outcomes such as improved quality of life, empowerment, and daily mental physical and social functioning	The extent to which patient-reported feedback is integrated into personalised care plans and decision-making
Technological	The use of digital tools and platforms, such as ePROMs and patient portals, which facilitate patient engagement in care assessment	Patient experiences with e-health tools, including usability, accessibility, and reporting experiences and outcomes	The digital infrastructure and interoperability of EHRs that support the capture, analysis and sharing of patient feedback	How e-health solutions influence clinical decision-making and care evaluation	The extent to which digital systems support consistent care assessment approaches across settings

**Fig. 2:**
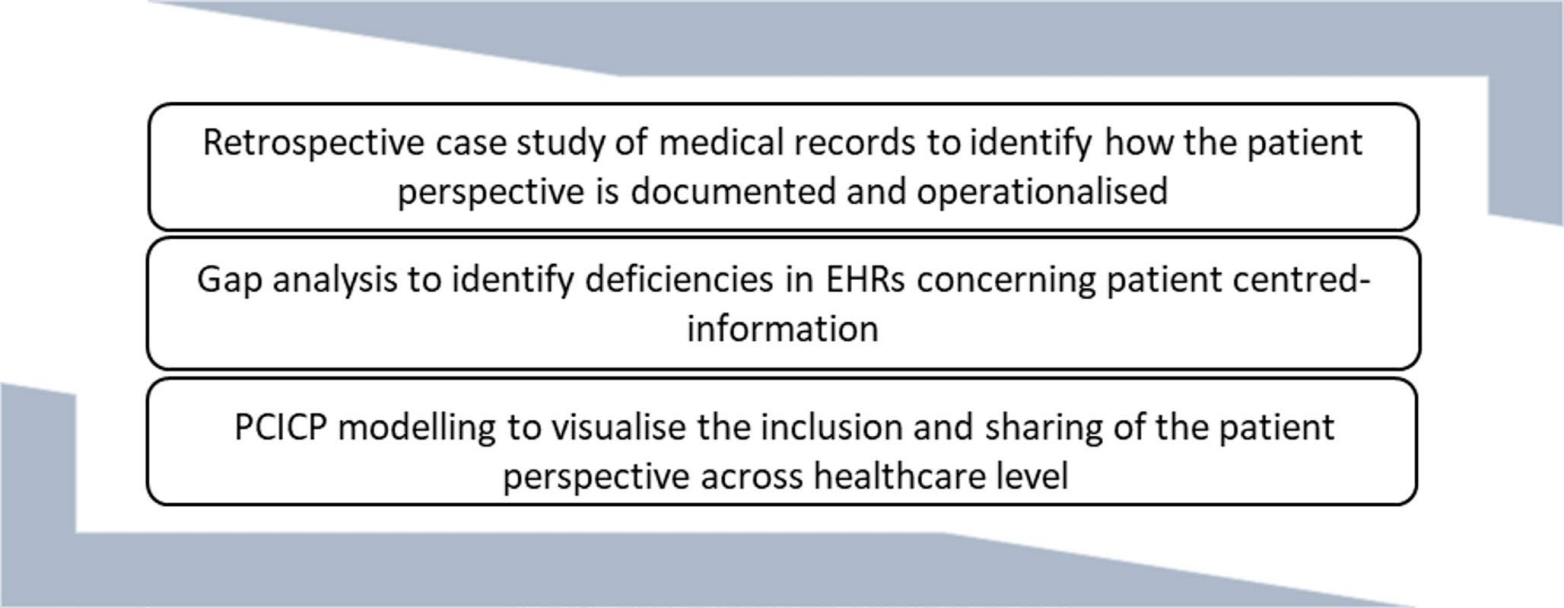
Overview of the three-phased case study design

To enhance validity, results from the literature review were compared with data from the Cassidy et al. case study [70] to determine how existing care assessment models aligned with, or failed to align with, patient-defined priorities. The case study was a mixed methods study conducted in three phases (Fig. 2):

Published longitudinal medical records and patient–provider interactions were analysed and then compared against the PECIA mapping using a framework-based matrix. All procedures were conducted in accordance with ethical guidelines and regulations.

### Results

**Fig. 3:**
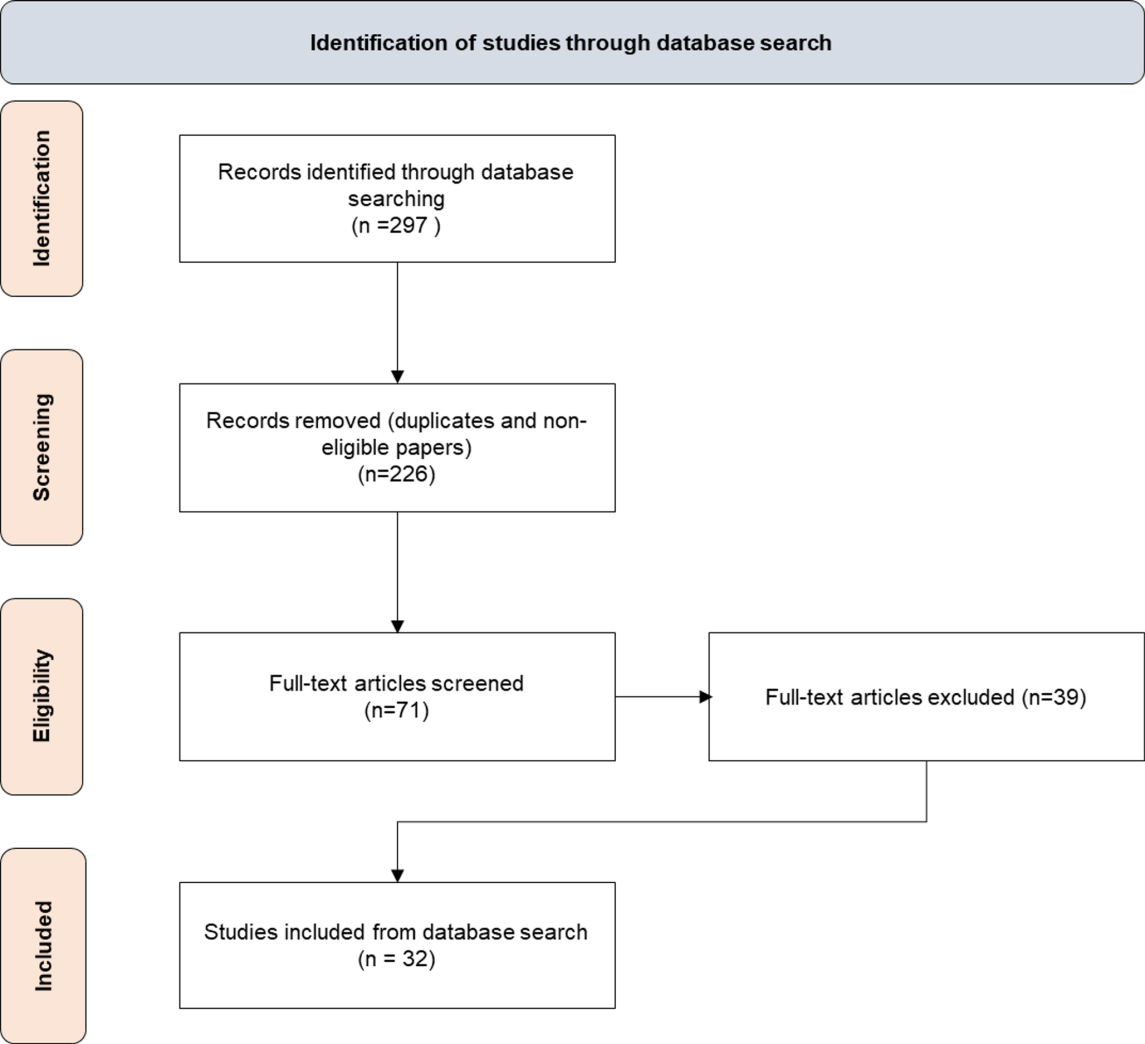
Prisma diagram of the study selection process

The literature review identified 297 papers. After screening titles and abstracts, 228 were excluded, leaving 69 for full-text review. Of these, 32 met our inclusion criteria. Figure 3 presents a detailed overview of the screening process.

### Study characteristics

#### Macro-level findings

The included studies examined diverse aspects of patient-centred care assessment across macro, meso, micro, and technological levels. A majority (81%) addressed multiple levels, indicating that the literature recognises the complexity of patient-centred care. Three (9%) focused exclusively on macro-level interventions, such as the implementation of accountable care organisations or transitions to patient-centred medical homes [74–76], with assessments commonly reporting patient satisfaction, service utilisation, and cost-effectiveness. This indicates a disconnect between national policy goals and patient-defined well-being, often relying on condition-specific metrics rather than broader quality-of-life measures [77–79].

#### Meso-level findings

At the meso level, many studies used standardised PROMs and PREMs to assess service delivery, mainly focusing on patient interactions, communication, safety, and satisfaction. While most studies confined patient roles to evaluating outcomes, only seven (22%) engaged patients more extensively in designing evaluation processes and defining outcome measures [74, 77, 79–83]. The limited extent of patient involvement throughout the development and evaluation of outcomes indicates a constraint on the potential for organisational-level metrics to genuinely reflect patient priorities. This restricts the *Participation* and *Alignment* components of care assessment (Table 4).

Eight studies involved non-clinical professionals like social workers to enhance care delivery perspectives [58, 78, 81, 84–88]. One study included incentives, such as travel reimbursement, to encourage patient participation [89], indicating potential structural barriers to equitable and comprehensive care evaluation.

#### Micro-level findings

Mental health outcomes were addressed in sixteen studies (50%), assessing aspects like emotional well-being and anxiety [58, 77, 79, 81, 82, 85–88, 90–96]. Twelve studies (38%) examined care for patients with complex health conditions requiring coordination across services and providers [58, 74, 75, 78, 81, 85, 90, 94, 97–100].

Although satisfaction surveys were common, they typically lacked measures of coordination and communication, suggesting a critical omission in evaluating integrated care. Ten studies identified limited alignment of long-term goals across organisations as a barrier to integrated care [58, 74, 79, 82, 83, 85, 87, 91, 92]. Furthermore, patient-centred outcomes, such as safety perceptions, practical support, or the influence of feedback on policy, were rarely examined [79, 81, 87, 98]. Nonetheless, 18 studies (56%) emphasised dimensions such as dignity, self-management, and social and emotional well-being as crucial for patient-centred care [58, 78, 79, 83, 87, 91, 96, 98, 99], aligning with the WHO’s multidimensional Quality of Life (QoL) definition described in the Introduction [24].

#### The technological dimension of care evaluation

Technological dimensions were addressed in 17 studies (53%), reflecting the growing importance of digital tools and data collection in patient-centred care assessment [74, 75, 83, 85, 88, 90, 92, 94, 95, 97–99, 101, 102]. Despite this technological adoption, 69% highlighted the lack of robust frameworks and comprehensive patient-centred outcome measures. Over half (56%) of the studies used outcomes not designed for standardised collection or EHR integration, complicating automated evaluation, decision support, and long-term tracking [58, 74, 78, 79, 81, 83, 86–89, 91, 93, 96–99, 103, 104]. This poses a fundamental challenge to scalability and automation of care evaluation. Without standardized, EHR-compatible measures, the potential of digital tools for real-time decision support and long-term tracking remains limited, indicating low *Context*, *Impact*, and *Alignment* (Table 4).

Few studies considered the healthcare provider’s perspective on the challenges of incorporating patient-reported data [83, 94, 95, 99], citing alignment issues between clinical workflows and patient-reported information, uncertainty around data interpretation, and lack of actionable integration in routine decision-making. This underscores an implementation gap between collecting patient data and actively using it to drive clinical decisions.

Further details are provided in Table 3.

**Table 3:** Characteristics and aims of the studies included in the literature review

References		Publication year	Country	Aim
Lancaster et al. [99].	The Use and Effects of Electronic Health Tools for Patient Self-Monitoring and Reporting of Outcomes Following Medication Use: Systematic Review	2018	Canada	Assess the impact of interventions on care quality
Knowles et al. [83]	Participatory co-design and normalisation process theory with staff and patients to implement digital ways of working into routine care: the example of electronic patient-reported outcomes in UK renal services	2021	United Kingdom	Assess the impact of interventions on care quality
Stehlik et al. [95]	Implementation of Real-Time Assessment of Patient-Reported Outcomes in a Heart Failure Clinic: A Feasibility Study	2017	United States	Incorporate patient experiences into intervention development and assessment
Davis et al. [88]	Paving the Way for Electronic Patient-Centered Measurement in Team-Based Primary Care: Integrated Knowledge Translation Approach	2022	United States	Incorporate patient experiences into intervention development and assessment
Clarke et al. [102]	Promoting integrated care in prostate cancer through online prostate cancer-specific holistic needs assessment: a feasibility study in primary care	2020	United Kingdom	Assess the impact of interventions on care quality
Strachna et al. [101]	Case study of the integration of electronic patient-reported outcomes as standard of care in a head and neck oncology practice: Obstacles and opportunities	2021	United States	Assess the impact of interventions on care quality
Cross et al. [77]	Developing a Theory of Change for a Digital Youth Mental Health Service (Moderated Online Social Therapy): Mixed Methods Knowledge Synthesis Study	2023	Australia	Incorporate patient experiences into intervention development and assessment
O’Loughlin et al. [100]	Review of patient-reported experience within Patient-Centered Medical Homes: insights for Australian Health Care Homes	2017	Australia	Assess the impact of interventions on care quality
Theis et al. [74]	Meaningfulness, feasibility, and usability of quality-of-care measures for maternal and infant health: A structured mixed-methods review	2024	United States	Assess the impact of interventions on care quality
Wilson et al. [75]	The impacts of accountable care organisations on patient experience, health outcomes and costs: a rapid review	2020	United States	Assess the impact of interventions on care quality
Westman et al. [103]	Patient-reported perceptions of care after the introduction of a new advanced cancer nursing role in Sweden	2019	Sweden	Assess the impact of interventions on care quality
Morales-Asencio et al. [78]	Living with chronicity and complexity: Lessons for redesigning case management from patients’ life stories - A qualitative study	2016	Spain	Incorporate patient experiences into intervention development and assessment
Carr et al. [80]	Co-design of a patient experience survey for arthritis central intake: an example of meaningful patient engagement in healthcare design	2019	Canada	Identify patient-centred quality indicators
Cook et al. [76]	Supporting Medical Home Transformation Through Evaluation of Patient Experience in a Large Culturally Diverse Primary Care Safety Net	2016	United States	Assess the impact of interventions on care quality
Virdun et al. [96]	Generating key practice points that enable optimal palliative care in acute hospitals: Results from the OPAL project’s mid-point meta-inference	2021	Australia	Assess the impact of interventions on care quality
Marques et al. [97]	Patient-centered care for patients with cardiometabolic diseases: An integrative review	2021	Portugal	Identify patient-centred quality indicators
Kelly et al. [58]	Measures for the integration of health and social care services for long-term health conditions: a systematic review of reviews	2020	United Kingdom	Identify patient-centred quality indicators
Albarqi [85]	Assessing the Impact of Multidisciplinary Collaboration on Quality of Life in Older Patients Receiving Primary Care: Cross Sectional Study	2024	Saudi Arabia	Assess the impact of interventions on care quality
Aryasinghe et al. [81]	Improving the maternity experience for Black, African, Caribbean and mixed-Black families in an integrated care system: a multigroup community and interprofessional co-production prioritisation exercise using nominal group technique	2024	United Kingdom	Assess the impact of interventions on care quality
Schmid et al. [93]	Patient perspectives on health care models in cardiac surgery: a qualitative evaluation	2024	Germany	Assess the impact of interventions on care quality
Shortell et al. [94]	A Multilevel Analysis of Patient Engagement and Patient-Reported Outcomes in Primary Care Practices of Accountable Care Organizations	2017	United States	Assess the impact of interventions on care quality
Sand-Svartrud et al. [92].	Associations between quality of health care and clinical outcomes in patients with rheumatic and musculoskeletal diseases: a rehabilitation cohort study	2022	Norway	Assess the impact of interventions on care quality
Barker et al. [90]	Values-Based Interventions in Patient Engagement for Those with Complex Needs	2020	United States	Assess the impact of interventions on care quality
Pellowski et al. [89]	You must leave but I didn’t want to leave”: qualitative evaluation of the integration of ART into postnatal maternal and child health services in Cape Town, South Africa	2020	South Africa	Assess the impact of interventions on care quality
van Ens et al. [87]	Place-Based FACT: Treatment Outcomes and Patients’ Experience with Integrated Neighborhood-Based Care	2024	Netherlands	Assess the impact of interventions on care quality
O’Donnell et al. [79]	Enabling public, patient and practitioner involvement in co-designing frailty pathways in the acute care setting	2019	Ireland	Incorporate patient experiences into intervention development and assessment
Valentine et al. [86]	Formative evaluation prior to implementation of a brief treatment for posttraumatic stress disorder in primary care	2023	United States	Assess the impact of interventions on care quality
Roos et al. [84]	Evaluation of an integrated care pathway for out-of-hospital treatment of older adults with an acute moderate-to-severe lower respiratory tract infection or pneumonia: protocol of a mixed methods study	2023	Netherlands	Assess the impact of interventions on care quality
Freeman et al. [91]	Development and evaluation of the Rural and Northern Community Focused Model of COPD Care (RaNCoM)	2023	Canada	Assess the impact of interventions on care quality
Højen et al. [82]	Development of A structured integrated post-Pulmonary Embolism care model: The Attend-PE model	2024	Denmark	Incorporate patient experiences into intervention development and assessment
Palladino et al. [98]	Evaluation of the North West London Diabetes Foot Care Transformation Project: A Mixed-Methods Evaluation	2022	United Kingdom	Assess the impact of interventions on care quality
Northwood et al. [104]	Care coordination of older adults with diabetes: A scoping review	2023	United Kingdom	Assess the impact of interventions on care quality

### Silent narratives: *Revealing the missing patient* case study

The *Revealing the Missing Patient* case study [70] examined how patient-provided information is often omitted in HISs by reviewing 1,117 records from general practice, hospital care, emergency departments, mental health services, social and home care. Participants had multiple diagnoses, including depression or personality disorders and physical illnesses such as cancer. Despite frequently discussing care preferences, health goals, life events, and social needs, only 121 records (9%) documented non-clinical, patient-provided data.

In some cases, the lack of patient-centred documentation led to interventions that conflicted with patients’ goals. For example, a patient with anxiety, depression, and suicidal ideation following a cancer diagnosis was given in-home nursing care, ignoring the patient’s request for assistance in reconnecting socially by visiting a local café with the help of a mobility aid. The case study also highlighted disconnected records and poor information exchange between providers, which led to duplicated services and inefficient care transitions.

These findings mirror the literature review, reinforcing the conclusion that there are significant gaps in integrating patient perspectives in care assessments, especially for individuals navigating complex mental and physical health challenges. EHRs fail to capture the subjective health and life goals that truly define patient-centred care, resulting in patient needs and goals being absent from system-level planning. Table 4 summarises the analysis.

**Table 4:** Summary of the literature review and case study findings mapped to PCICP levels and PECIA framework components

PCICP level	Findings from literature review	PECIA component literature review	Findings from case study	PECIA component case study
Macro	Policy goals often conflict with patient-defined health priorities. Aggregated PROMs are used, but patient-defined QoL is rarely prioritised. The evaluation focuses on costs and clinical efficiency.	Lack of Participation and Alignment. Experience, Context and Impact N/A	Patient needs and goals are absent from system-level planning. EHRs prioritise biomedical data, leaving out subjective health and life goals. Policies do not reflect real patient experiences.	Lack of Participation and Alignment. Experience, Context and Impact N/A
Meso	PROMs/PREMs are commonly used in quality improvement but seldom inform evaluation design, include long-term goals across organisations, involve non-clinical professionals such as social workers, engage patients in designing evaluation processes and defining relevant outcome measures, or consider the healthcare perspective on patient-centred care assessment.	A somewhat higher degree of Participation and Experience. Low degree of Context, Impact and Alignment	Gaps in organisational documentation practices hinder the sharing of patient perspectives across systems, compromising continuity of care. Outpatient care services often lack access to information from other providers, making it challenging to deliver appropriate daily care or plan further services	Low degree of Participation and Experience. Lack of Context, Impact and Alignment
Micro	PROMs/PREMs assess individual care but overlook evolving needs. Emotional, practical, identity-based aspects valued by patients are poorly represented in standardised tools, and the influence of feedback on policy is rarely examined	A somewhat higher degree of Participation, Experience, Impact, Context and Alignment	Non-clinical notes contained patients’ life goals, social context, emotional state, and priorities. Most of the data were retrospective and did not include planned activities, appointments, or care pathways.	Low degree of Participation and Experience. Lack of Context, Impact and Alignment
Techno	Digital tools support PROMs and PREMs collection but lack interoperability. Patient experiences and outcomes were not designed for structured collection or integration into EHRs, complicating automated evaluation, real-time decision support, and long-term tracking.	Low Participation, Experience, Context, Impact and Alignment	Inconsistent coding systems, lack of interoperability inadequate regulation of data sharing among care providers, and a lack of standardised methods for documenting and sharing patient perspectives hinder a common understanding of when, how, or why patient perspectives should be included.	Low Participation, Experience, Context, Impact and Alignment

## Discussion

This study examined the methodologies for incorporating patient perspectives into care assessment across healthcare levels. It highlights critical gaps and reinforces research findings that care assessments risk excluding what matters most without systematically capturing patient-defined priorities. At the macro level, current evaluation frameworks frequently emphasise clinical effectiveness and cost, neglecting individual experiences and quality of life. At the meso level, PROMs and PREMs often do not fully capture patients’ evolving experiences and values, instead mirroring organisational and clinical routines. At the micro level, assessment frameworks rarely consider the subjective dimensions valued by patients, such as autonomy, dignity, and emotional support.

Our findings, summarised in Table 5, confirm that existing tools are often siloed rather than integrated. While PROMs and PREMs capture dimensions of outcomes and experiences, our study demonstrates they fall short in addressing patient-defined priorities and contextual factors crucial for those with complex needs. These limitations underscore the need for additional measures that holistically capture what matters to patients. To bridge these gaps, we propose Patient-Reported Integrated Measures (PRIMs), where *integrated* implies the multidimensional nature of patient-centred care in assessment processes, allowing for a structured representation of the patient’s voice, even when not directly involved in decision-making. PRIMs complement PROMs and PREMs, but uniquely assess the integration of patient-defined outcomes into the broader context of healthcare delivery and policy alignment, helping to translate broad frameworks such as the WHO’s Comprehensive Mental Health Action Plan and the OECD Better Life Initiative into actionable metrics within health systems. For instance, while PROMs may measure symptom reduction, PRIMs can track personal health goals like socialising with friends, highlighting gaps between provided care and patient needs.

Grounding PRIMs in the PCICP framework enables a practical approach and supports systematic integration of patient perspectives across healthcare levels, enabling the evaluation of patient experiences alongside clinical outcomes and aligning care with national guidelines. Furthermore, the framework can support the identification of gaps in current evaluations and guide healthcare professionals, providers, and national institutions in developing new multidimensional, patient-centred indicators for more responsive evaluations in both clinical and non-clinical care.

### Using prims in care assessments

**Fig. 4:**
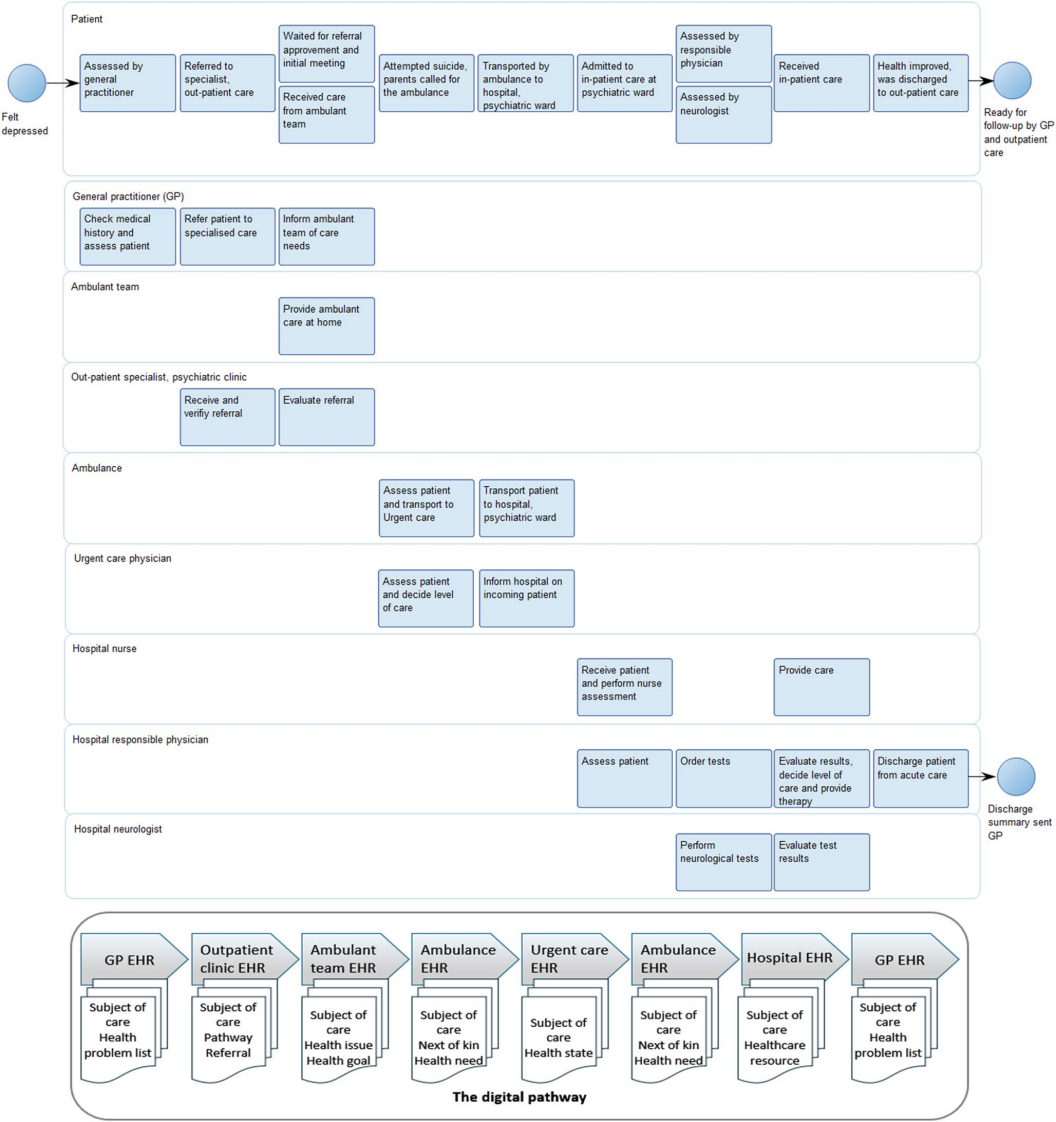
Patient-centred, integrated care pathway (PCICP) for a patient’s co-occurring mental and physical illness. Modelled using Business Process Modelling Notation (BPMN) and QualiWare Lifecycle Manager 10.3

To illustrate the implementation of PRIMs in practice, we discuss a PCICP-informed case involving a patient navigating care for cancer, anxiety, and depression across multiple services. This pathway encompasses personal experience (micro level), provider coordination (meso level), national guidelines (macro level), and data interoperability through electronic health records (technological level), as illustrated in Fig. 4. It includes key actors such as GPs, psychiatric services, hospital teams, and home care nurses, along with decision points and the flow of digital documentation. Within this model, PRIMs assess how well care activities align with national objectives and the patient’s lived experience.

At the micro level, the patient’s goals, such as reconnecting socially and managing anxiety in daily life, were not addressed despite having been communicated. At the meso level, poor coordination between the general practitioner (GP) and the mental health team resulted in fragmented care. Additionally, national mental health policies that emphasise early intervention were not effectively implemented at the macro level. These issues were further complicated by fragmented electronic health records and gaps in data exchange among providers.

On the meso level, PRIMs could monitor whether the healthcare providers are equipped and incentivised to include non-clinical patient perspectives in decisions and that are shared across disciplines and organisations, or whether their organisational practices genuinely integrate patient-defined outcomes into planning and evaluation. Regardless, these indicators should not be confined to one-time measurements. Longitudinal tracking of patients’ evolving mental and physical health status, functional capacities, and psychosocial well-being is essential.

Table 5 illustrates how PRIMs could complement existing PROMs and PREMs across the care continuum.

**Table 5:** Examples of PROMs, PREMs and prims across PCICP levels

PCICP level	PROM example	PREM example	PRIM example
Macro	Aggregated mental health status data, such as psychological well-being across populations [17]	System-level patient experience surveys, such as unmet care needs [16]	Percentage of patients reporting alignment between national healthcare policies and guidelines and their care experience
Meso	Symptom improvement in chronic diseases, such as functional status changes after an intervention [17]	Patient experiences with care coordination and staff responsiveness [17]	Proportion of patient-defined care goals integrated into care planning and reviewed with patients and their multidisciplinary team
Micro	Ability to carry out everyday physical activities [7]	Degree of confidence to manage one’s health [7]	Patients’ experience of support to manage their health problems, such as by telehealth or ambulant teams, while waiting for specialised care provision
Techno	Electronic patient-reported outcomes (ePROMs) for assessing patient self-monitoring and symptom tracking [17]	Patient experiences of digital interfaces for self-reporting [22]	Degree of inclusion and consistency of patient-defined goals documented across electronic health records

### Operationalising prims

One approach to PRIMs development could be to adapt development steps for other patient-centred indicators [105], embedding the PCICP levels and PECIA components. Following this approach, the purpose and scope should first be defined to specify what PRIMs will be used for (research, quality improvement, or service evaluation), whether they are generic or disease-specific, and at which level (macro, meso, or micro). At the macro level, Participation could include feedback on the degree to which national policies include structured patient input, *Experience* and *Alignment* could assess whether system-level policies reflect patient-defined priorities, and *Impact* could examine whether including patient perspectives in policies yields observable effects over time. Additionally, a literature review can further inform indicator generation, with resulting candidate indicators grouped into PRIMs-aligned domains. Patients and relevant stakeholders should be involved throughout development, piloting, and evaluation. After constructing a pilot version, formally test reliability, validity, and ease of understanding; then eliminate poorly performing items using appropriate psychometric methods.

Collected through digital tools, PRIMs could be available as electronic PRIMs (ePRIM), in a standardised format so they can be shared across EHR systems, eHealth solutions and national health registries, making patient goals, needs, and priorities visible from point of care to policy and ensuring measurement reflects what matters to patients. Existing international standards like ISO 13940:2015, Health informatics – System of concepts [106], which offers a standardised system of concepts for continuity of care, could be used as a structured vocabulary. While originally rooted in clinical workflows, key concepts in the standards, such as *Health Goal* and *Non-ratified Healthcare Information* could be adapted to describe patient-defined priorities in a structured format, enabling the integration of PRIMs into EHR systems, facilitating longitudinal tracking and improving interoperability among organisations. Additionally, PRIMs could also be used to evaluate how well patient-generated data is recorded and reflected in clinical decision-support systems, strengthening the technological infrastructure for patient-centred, integrated care.

## Challenges

PRIMs present practical and ethical challenges, including barriers to sustained patient engagement, time constraints, workforce limitations, and complex patient needs. There are also concerns about privacy and data governance. Aligning patient-defined measures across healthcare levels remains challenging due to differing priorities and operational constraints. To mitigate these challenges, the development and implementation of PRIMs must be supported by robust ethical frameworks, promoting patient and family involvement in care evaluation, informed consent, cross-sector data sharing, confidentiality, and equitable participation for marginalised groups.

## Policy and practice implications

To strengthen the performance of health systems and ensure that care is more closely aligned with what matters to patients, we propose that national quality frameworks should be expanded to incorporate Patient-Reported Integrated Measures (PRIMs) alongside existing PROMs and PREMs in health information systems. This would offer a fuller picture of care quality across macro, meso and micro dimensions, including the collection and use of longitudinal data, especially for individuals living with multiple long-term conditions, to help track shifting priorities and needs over time. Embedding PRIMs into health information systems will enable structured documentation and active use of patient-defined goals in both care planning and service evaluation. Furthermore, interdisciplinary training is essential, ensuring that professionals across healthcare levels and disciplines consistently understand how to interpret PRIMs and apply them meaningfully in decision-making. Together, these steps ensure that patient perspectives shape how care is measured and improved. In doing so, quality development becomes not just a technical exercise but one rooted in the lived realities of those the system is meant to serve.

## Conclusion

This study examined how patient perspectives are incorporated into care evaluation across macro, meso, micro and technological domains. The findings indicate an ongoing disconnect between how healthcare quality is evaluated and what matters to patients, particularly those with complex health needs. Furthermore, our findings reveal ongoing misalignment between system-level evaluation practices and patient-defined outcomes, especially for individuals with complex physical and mental health needs.

Building on existing PROMs and PREMs, Patient-Reported Integrated Measures (PRIMs) extend measurement to include what patients themselves define as meaningful and situate these priorities within clinical practice, organisational routines and policy objectives, consistent with the PCICP framework. PRIMs are intended as a structured, multi-level approach that links individual goals with service delivery and governance and can be captured and used in digital systems.

PRIMs are presented here as a conceptual contribution that complements, rather than replaces, current instruments. Implementing PRIMs will demand technological, organisational, cultural, regulatory and ethical innovation. This shift is necessary if future reforms are to be genuinely patient-centred rather than system-driven. In doing so, PRIMs aim to bridge the gap between institutional metrics and patient-centred, integrated care. In short, advancing healthcare should ensure we do not *leave patients behind*, but move forward with them.

## Future research

Further exploration of the practical development, implementation, and validation of PRIMs within healthcare systems is required. Additionally, longitudinal studies would aid in assessing their validity and reliability across diverse healthcare contexts and patient populations, thereby supporting the transition from theoretical constructs to meaningful indicators for use in healthcare practice.

## Limitations

This paper acknowledges several limitations. Given the study’s theoretical and review-based nature, findings are constrained by the absence of primary data collection. Limiting inclusion to open-access, peer-reviewed articles and excluding grey literature may have introduced selection and publication bias, reducing representativeness and generalisability. Furthermore, the complexity of healthcare contexts, particularly regarding integrated care for individuals with complex health issues, necessitates context-specific adaptation of the proposed framework. In addition, PRIMs have not yet undergone real-world implementation or field validation. Operational feasibility therefore remains to be demonstrated.

## Appendix A. Study protocol

### Search strings

The search strings were all limited to open-access, peer-reviewed articles published in English between January 1, 2015, and January 1, 2025.

#### PubMed

*(Health Care Quality, Access, and Evaluation[MeSH Major Topic]) AND ((integrated care[Title/Abstract]) OR (coordinated care[Title/Abstract]) OR (integration[Title/Abstract]) OR (coordination[Title/Abstract])) AND (("patient engagement"[Title/Abstract]) OR ("patient involvement"[Title/Abstract]) OR ("patient participation"[Title/Abstract]) OR ("co-design"[Title/Abstract]) OR ("co-production"[Title/Abstract]) OR ("co-development"[Title/Abstract])) AND ((evaluation[Title/Abstract]) OR (assessment[Title/Abstract])) AND ((measure*[Title/Abstract]) OR (performance[Title/Abstract]) OR (indicator[Title/Abstract]) OR (outcome[Title/Abstract]) OR (impact[Title/Abstract])).*

#### Google Scholar

*(health and quality and ("integrated care" or "coordinated care" or integration or coordination) AND ("patient engagement" OR "patient involvement" OR "patient participation" OR co-design OR co-production OR co-development) and (evaluation or assessment) and (measure* or performance or indicator or outcome or impact).*

#### Web of Science

*AB* = *(health) and AB* = *(quality) and (AB* = *("integrated care") or AB* = *("coordinated care") or AB* = *(integration) or AB* = *(coordination)) and (AB* = *("patient engagement") or AB* = *("patient involvement") or AB* = *("patient participation") or AB* = *(co-design) or AB* = *(co-production) or AB* = *(co-development)) and (AB* = *(evaluation) or (AB* = *(assessment)) and (AB* = *(measure*) or AB* = *(performance) or AB* = *(indicator)) or AB* = *(outcome) or AB* = *(impact)).*

### Eligibility criteria

**Table:**

Criterion	Inclusion	Exclusion
Publication type	Peer-reviewed, open access	Not peer-reviewed, not available via open access
Language	English	Non- English
Publication date	January 1, 2015 and January 1, 2025	Published outside this date range
Patient-centred focus	Explicit focus on patient-centred care assessment in healthcare	Studies focusing only on provider, organizational, or administrative perspectives, disease outcomes, medical treatments, or interventions not linked to care assessment
Level of Care	Addressed multiple levels of care (e.g., primary, hospital, home/community care)	Focused exclusively on a single setting or professional group (e.g., only hospital, only GPs, only nurses
Study type	Empirical or conceptual studies (quantitative, qualitative, mixed methods); includes theme-based outcome studies	Protocols for planned interventions with no findings and studies exclusively addressing patient involvement in service design
Assessment scope	Assessment of care processes, outcomes, impact, quality, or use of indicators or frameworks for evaluation	Studies limited to patient involvement in service or intervention co-design (not care assessment)
Methodological Transparency	Describes specific assessment methods, measurement tools, or indicators	Describes care in general terms without clear methodology for evaluation

### Other criteria

1. Integrated/coordinated care focus (required)

The study explicitly addressed "integrated care," "coordinated care," and "multi-disciplinary teams (or synonyms for these expressions).

Exclusion: The study was excluded if integrated/coordinated care was only a minor or secondary theme and not the central focus.

2. Patient perspective (required)

The study incorporated patient perspectives in the discussion or evaluation of care quality.

- Patient perspectives included:
- Patient-reported outcomes (PROs)
- Patient-reported experience measures (PREMs)
- Qualitative accounts of patient feedback were used to inform or evaluate care quality.
- Direct consideration of patient views, preferences, or priorities in care assessment
- Patient involvement in shaping care assessment criteria, evaluation methods or similar (e.g., co-developing surveys, defining meaningful quality indicators)

Exclusion: The study was excluded if it only evaluated care from a provider, system, or policy standpoint without mentioning patient viewpoints.

If the study only discussed patient involvement in service/intervention design without involving patients in care assessment or assessing the outcomes of the care that results from the service/intervention, it was excluded.

3. Evaluation/measurement component (required)

The study had to propose, describe, or discuss an evaluation framework, model, tool, assessment approach, performance measure, indicators, or outcome related to integrated care.

Both conceptual and empirical studies were eligible.

Conceptual or theoretical models were included as long as they addressed the evaluation of integrated care from a patient-centred perspective.

Exclusion: If the study only discussed integrated care in general terms, without any reference to evaluation, measurement, or assessment, it was excluded.

4. Healthcare context (not mandatory)

- Studies conducted in healthcare systems serving populations with chronic or complex conditions were included
- Studies covering primary, secondary, or tertiary care settings in an integrated/coordinated model were included.

Not required for exclusion—a study was included if it met all mandatory inclusion criteria.

5. Type of care being assessed (not mandatory)

The study must specify the clinical problem or healthcare setting it assesses rather than only discussing healthcare broadly.

Not required for exclusion—a study was included if it met all mandatory inclusion criteria.

After evaluation, each study was categorised as follows:

- Included – Meets all inclusion criteria.
- Included with limitations – Lacks some preferred elements but still provides valuable insight.
- Excluded – Fails to meet one or more required criteria.
- Further review is needed – Ambiguous cases require discussion before making a final decision.

### Data extraction

Variables extracted from the full review articles:

1. Aim of the care assessment – What was the primary objective of the care evaluation in the study?
2. Study method used – What methods were used for the care evaluation (e.g., qualitative, quantitative, mixed methods)?
3. Stakeholders involved (other than patients) – Which groups (e.g., clinicians, policymakers, administrators) were included in the assessment process?
4. Level(s) of care assessment – At which level(s) was the assessment conducted? (Macro, meso, micro)
5. Patient involvement in the care assessment process. How patients were engaged in the care assessment What mechanisms were used to involve patients (e.g., surveys, focus groups, co-design workshops)?
6. Stage(s) of involvement – At what stage(s) in the evaluation process were patients engaged? (Planning & developing outcomes, evaluating outcomes, others)
7. Care assessment methodologies. Evaluation approach, framework, or tool used for care assessment – What approach, model, framework, or tool was used?
8. Type of care being assessed – What kind of care was being evaluated?
9. Was it a clinical intervention, such as a new treatment, a technical intervention, such as a health app or system, or something else?
10. What was the goal of the intervention?
11. Patient-centred outcomes mentioned – Does the study identify specific patient-centred outcomes? If so, what were they?
12. What additional outcomes (e.g., cost, efficiency, clinical effectiveness) were considered in care assessment? What measurement indicators were used to assess the described outcomes, both patient-centred and others.
13. Was only qualitative analysis conducted and observable measures extracted? (e.g., patient satisfaction ratings, engagement tracking, structured thematic coding of patient feedback).Differentiated structured indicators from patient-reported themes
14. Evidence for measurements and outcomes – What empirical or qualitative evidence sources supported the selected metrics and outcomes?
15. Was patient feedback integrated in the care assessment?
16. Use of patient feedback – How was patient feedback incorporated into the assessment? Did patient perspectives influence assessment criteria (e.g., shaping quality indicators, contributing to framework design)?
17. Standardisation of patient feedback – Was the patient feedback structured or standardised to allow integration into digital clinical decision-support systems?
18. Did the study explicitly mention concrete incentives, such as financial compensation, training, dedicated roles, or organisational support, provided to support the inclusion of patient perspectives in care assessment?
19. Identified gaps in existing assessments or outcomes used – Does the study explicitly highlight areas where current care assessments fall short?
20. Barriers to including patient perspectives in care assessment – What obstacles to patient involvement in care assessment were described?
21. Enablers for including patient perspectives in care assessment – What factors supporting successful patient involvement in care assessment were described in the paper?
